# Agricultural Origins of a Highly Persistent Lineage of Vancomycin-Resistant *Enterococcus faecalis* in New Zealand

**DOI:** 10.1128/AEM.00137-19

**Published:** 2019-06-17

**Authors:** Rowena Rushton-Green, Rachel L. Darnell, George Taiaroa, Glen P. Carter, Gregory M. Cook, Xochitl C. Morgan

**Affiliations:** a Department of Microbiology and Immunology, University of Otago, Dunedin, New Zealand; b Department of Biochemistry, University of Otago, Dunedin, New Zealand; c Doherty Institute, University of Melbourne, Parkville, Australia; d Maurice Wilkins Centre for Molecular Biodiscovery, University of Auckland, Auckland, New Zealand; Centers for Disease Control and Prevention

**Keywords:** *Enterococcus*, VRE, antimicrobial, bacitracin, *faecalis*, *faecium*, genomics, phylogeny, vancomycin

## Abstract

Enterococcus faecalis and Enterococcus faecium are human and animal gut commensals. Vancomycin-resistant enterococci (VRE) are important opportunistic pathogens with limited treatment options. Historically, the glycopeptide antibiotics vancomycin and avoparcin selected for the emergence of vancomycin resistance in human and animal isolates, respectively, resulting in global cessation of avoparcin use between 1997 and 2000. To better understand human- and animal-associated VRE strains in the postavoparcin era, we sequenced the genomes of 231 VRE isolates from New Zealand (NZ; 75 human clinical, 156 poultry) cultured between 1998 and 2009. E. faecium lineages and their antibiotic resistance carriage patterns strictly delineated between agricultural and human reservoirs, with bacitracin resistance ubiquitous in poultry but absent in clinical E. faecium strains. In contrast, one E. faecalis lineage (ST108) predominated in both poultry and human isolates in the 3 years following avoparcin discontinuation. Both phylogenetic and antimicrobial susceptibility (i.e., ubiquitous bacitracin resistance in both poultry and clinical ST108 isolates) analyses suggest an agricultural origin for the ST108 lineage. VRE isolate resistomes were carried on multiple, heterogeneous plasmids. In some isolate genomes, bacitracin, erythromycin, and vancomycin resistance elements were colocalized, indicating multiple potentially linked selection mechanisms.

**IMPORTANCE** Historical antimicrobial use in NZ agriculture has driven the evolution of ST108, a VRE lineage carrying a range of clinically relevant antimicrobial resistances. The persistence of this lineage in NZ for over a decade indicates that coselection may be an important stabilizing mechanism for its persistence.

## Abstract (precis)

Historical antimicrobial use in NZ agriculture has driven the evolution of ST108, a VRE lineage carrying a range of clinically relevant antimicrobial resistances. The persistence of this lineage in NZ for over a decade indicates that coselection may be an important stabilizing mechanism for its persistence.

## INTRODUCTION

Enterococci are commensal colonizers of humans and animals and important opportunistic pathogens in nosocomial infection (1–8). Enterococcus faecalis and E. faecium are the most common human-associated enterococci, causing ∼66,000 infections per year in the United States alone, approximately one-third of which are antibiotic resistant (9). Enterococci are intrinsically resistant to many classes of antibiotics and easily acquire further resistance (10). For this reason, strains that have acquired vancomycin resistance are particularly difficult to treat (6, 11, 12). Furthermore, enterococci can serve as a reservoir for resistance determinants that are transferable to other clinically relevant bacteria (13, 14).

Vancomycin is a clinically significant glycopeptide antibiotic. Historically, two factors have been causatively linked to the rise of vancomycin-resistant enterococci (VRE). The first is the clinical overuse of vancomycin, and the second is the agricultural use of avoparcin, a glycopeptide structurally similar to vancomycin (7, 15, 16). Avoparcin use has been broadly discontinued since the late 1990s as a consequence of its link to the development of animal-associated VRE (17–20). These preventative actions effectively lowered the prevalence of animal-associated VRE in many locations, although VRE were reported to persist within agricultural settings for up to 5 years after avoparcin use (18, 21–25).

Within New Zealand (NZ), avoparcin was historically used primarily for growth promotion in poultry. Legislation was introduced in 1997 requiring veterinary prescriptions for antibiotic use on livestock (26), and avoparcin was discontinued from 2000. Other classes of antibiotics, such as the antimicrobial peptide bacitracin and the macrolide tylosin, are still commonly used in the poultry industry (27, 28). Previous studies surveyed NZ VRE isolates after avoparcin discontinuation to observe the effect of its withdrawal on the prevalence of VRE species and lineages in both clinical and agricultural settings (29–31). Pulsed-field gel electrophoresis (PFGE) analysis of the VRE isolates showed that vancomycin-resistant E. faecium populations had high genetic diversity but maintained a *vanA* genotype (29, 30). In contrast, a highly prevalent group of VanA-type E. faecalis was identified in both clinical and poultry isolates. E. faecium was the most prevalent VRE species in most countries during this time period, so the high prevalence of vancomycin-resistant E. faecalis in NZ during this time period presented a notable contrast (6, 20, 25, 32–34). The majority of these NZ VRE isolated from poultry were also erythromycin and bacitracin resistant (29, 30). Analysis of the plasmids found in poultry VRE isolated during the period from 2000 to 2001 showed the carriage of both *vanA* and *ermB*, indicating probable coselection of resistance, as well as plasmid transfer between enterococcal species (29).

Based on these findings, a strong phylogenetic link was hypothesized between NZ poultry and clinical vancomycin-resistant E. faecalis isolates during this sampling period (2000 to 2003). However, this conclusion could not be definitively drawn from the PFGE analysis available at the time of investigation (29–31). We sought here to test this hypothesis by performing whole-genome sequence analysis on this collection of historical VRE isolates, as well as additional clinical NZ VRE strains that were isolated between 1996 and 2009. We used this sequencing data to generate detailed phylogenies of vancomycin-resistant E. faecalis and E. faecium. We also examined the genomic distribution of antibiotic resistance genes within and between isolates. The results of this study showed a close relationship between clinical and agricultural isolates of ST108, a highly persistent and clinically significant lineage of VanA-type E. faecalis.

## RESULTS

To generate a detailed phylogeny of VRE in New Zealand (NZ), clinical and poultry isolates from previous studies (29, 30) or collected by the Institute of Environmental Science and Research (ESR) prior to 2010 were sequenced using Illumina whole-genome sequencing (see Table S1 and Fig. S1 in the supplemental material). This retrospective work focused on two of the most prevalent VRE species, E. faecalis (113 isolates) and E. faecium (116 isolates). Core genome phylogenies and antibiotic-resistance gene repertoires of VRE isolates, along with relevant metadata, were used to predict epidemiological relationships. The Nullarbor pipeline (35) and Gubbins (36) were used for multilocus sequencing typing (MLST) to generate core genome alignments and phylogenies, to correct alignments for recombination, and to identify resistomes (see Materials and Methods for further details).

### Vancomycin-resistant *E. faecium* strains from postavoparcin New Zealand are polyclonal and host specific.

**FIG 1:**
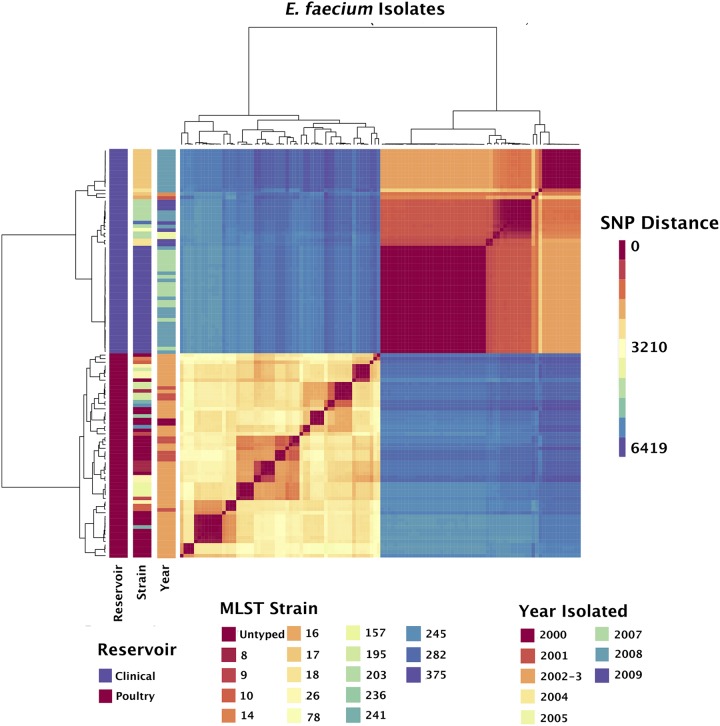
Pairwise SNP distances between the core alignments of vancomycin-resistant E. faecium isolates. Heatmap color indicates the number of pairwise SNPs between isolates. Isolates are clustered according to the number of shared SNPs. Vertical bars (left) indicate the sample reservoir, MLST strain, and year of isolation of each isolate. The core alignment comprises a clonal frame of 2,933,268 bp with 9,835 SNPs. The reference sequence is a clinical E. faecium strain E1 complete genome (NCBI RefSeq NZ_CP018065).

We used the assembled clinical E. faecium E1 genome as a reference to produce a recombination-corrected core alignment of the 116 E. faecium isolates (Fig. S2 and S3a). Two untyped phylogenetic outliers (ARL98-565 and ARL09-409) were removed to improve visualization of relationships between isolates, generating a core genome alignment with 9,833 variable sites (Fig. 1, Fig. S3b). The E. faecium pangenome comprised 7,571 genes, of which 1,585 were core genes.

E. faecium clinical (*n* = 59) and poultry (*n* = 57) isolates separated into phylogenetically distinct lineages with no overlapping multilocus sequence types, consistent with previous literature (37, 38). While the poultry isolates in this study were collected prior (2000 to 2003) to most clinical isolates, phylogenetic separation by host was robust across time. Clinical E. faecium were dominated by three multilocus sequence types. Poultry lineages were more diverse, with 10 unique multilocus sequence types, as well as 11 additional unique combinations of MLST alleles of unknown multilocus sequence types (Fig. 1). Despite greater heterogeneity, poultry isolates had slightly smaller pangenomes (4,877 poultry versus 5,563 clinical) and slightly more (1,666 poultry versus 1,563 clinical) core genes. Three pairs of clinical isolates, all from ST375, differed by fewer than eight single-nucleotide polymorphisms (SNPs), suggesting a close relationship (39). The observed separation of E. faecium isolates into reservoir-based lineages suggests a low frequency or complete lack of E. faecium movement between poultry and human hosts in NZ.

We used the BactDating R package (40) to estimate the divergence of poultry and clinical E. faecium lineages. The analysis suggested that within this data set, all clinical E. faecium isolates were descended from a common ancestor between 1971 and 1994 (95% credible interval), and all poultry E. faecium isolates were descended from a common ancestor between 1902 and 1972 (95% credible interval) (Table S2). The former estimate is consistent with the reported emergence of clinical VR E. faecium in 1988 (41) and subsequent introduction to NZ. The latter estimate may be consistent with ancestral poultry strains undergoing selective pressures such as other antibiotic exposure prior to acquisition of *vanA* genes. The mean rate of substitution μ was 2.02 substitutions per genome per year (1.13 to 3.28) with a standard deviation σ of 1.5 (0.73 to 2.7). We observed that the Tajima’s D value for E. faecium was −3.10 (*P* < 0.01), indicating recent purifying selection.

### Vancomycin-resistant *E. faecalis* isolates from the postavoparcin period are predominantly of the single multilocus sequence type, which colonizes multiple host species.

**FIG 2:**
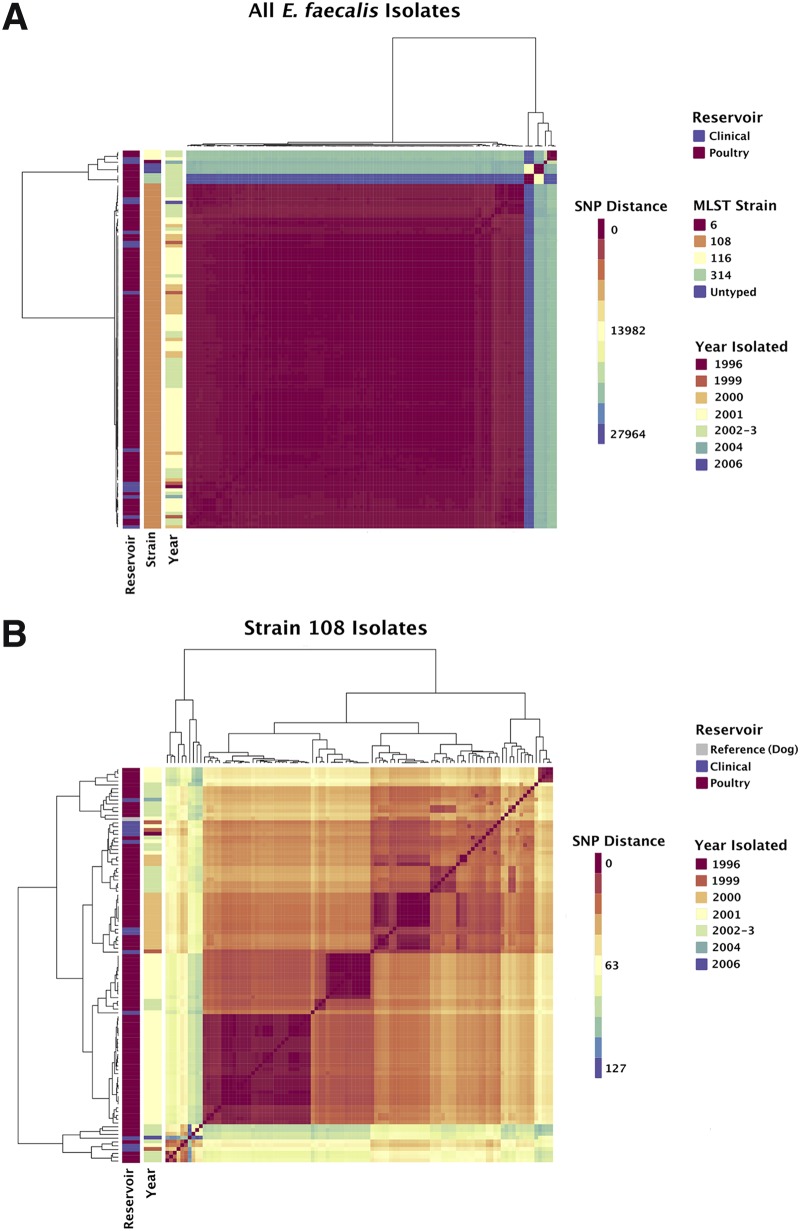
Pairwise SNP distances between the core alignments of vancomycin-resistant E. faecalis isolates. (A) All E. faecalis VRE isolates included in this study; (B) all E. faecalis ST108 isolates. The heatmap color indicates the number of pairwise SNPs between isolates. Isolates are clustered according to number of shared SNPs. Vertical bars (left) indicate the sample reservoir, MLST strain, and year of isolation of each isolate. For panel A, the reference genome was E. faecalis CLB21560. The core alignment comprised 3,111,017 bp with 42,064 SNPs. For panel B, the reference genome was the AR01/DG chromosome. The core alignment comprised 2,717,372 bp with 665 SNPs.

We used the assembled clinical E. faecalis CLB21560 genome as a reference to produce a recombination-corrected alignment of 113 E. faecalis genomic sequences spanning 1996 to 2006 (Fig. 2A). The E. faecalis pangenome had 4,925 genes, including 2,171 core genes. Notably, clinical and poultry isolates were interspersed within the E. faecalis phylogeny. Of all VR E. faecalis isolates, 103 (91.2%) belonged to a single lineage, multilocus sequence type 108 (ST108) (Fig. S4a). ST108 was of interest because most of the early clinical VRE isolates from NZ belonged to this lineage (Table S3). Furthermore, ST108 was the most common of all poultry-associated VRE lineages observed by Manson et al. (30). It was isolated from multiple farms on both the North and South Islands and from all three major NZ poultry suppliers (Table S1).

To achieve higher phylogenetic resolution within the ST108 clade, a recombination-corrected alignment of 103 ST108 genomes was constructed, using a complete PacBio-assembled ST108 genome, AR01/DG, as the reference (see Materials and Methods). This produced a core chromosomal alignment of 2,717,372 bp with 664 variable sites, with the isolates differing by a maximum of 127 SNPs, although the majority differed by fewer than 100 sites (Fig. 2B). Notably, both clinical and poultry isolates appear on the majority of sub-branches in the ST108 clade (Fig. 2B, Fig. S4b). This supports the original hypothesis that ST108 became prevalent in NZ in the late 1990s and has moved between human and poultry hosts. Analysis of E. faecalis with BactDating supported multiple chicken/clinical divergences throughout the 1990s (Fig. S5; Table S10) and suggested that all ST108 isolates had a common ancestor between 1926 and 1976 (95% credible interval). The mean rate of substitution μ was 1.47 substitutions per genome per year (0.89 to 2.14) with a standard deviation σ of 1.04 (0.58 to 1.64). This was supported by a Tajima’s D value of −2.34 (*P* < 0.05), which also supports recent purifying selection. This timeline could be consistent with emergence either prior to or immediately after the onset of avoparcin use in NZ in 1977.

In the pangenome of E. faecalis ST108 isolates, a total of 4,265 genes were detected, 2,502 of which were core genes (present in >95% of isolates). Isolates had from 2,681,276 to 2,717,372 bases of genome sequence present within the clonal frame. A total of 2,810 genes from the pangenome could be chromosomally assigned based on the reference genome. Of these, 2,473 were core genes, reflecting high chromosomal conservation across isolates. In contrast, plasmid-localized genes were less conserved (Fig. S6); of 175 plasmid-localized genes, only 26 (including the *vanA* operon) were core genes. Genes that could not be classified as either plasmid localized or chromosomal based on the reference genome (1,281 genes, 29 core genes) were the most poorly conserved across strains. Approximately two-thirds of these genes were detected in only one or two strains. Many of these may be assembly artifacts (Fig. S6).

### An abundant VRE clone carries multiple antimicrobial resistance plasmids.

One representative isolate (E. faecalis AR01/DG) from the E. faecalis ST108 lineage, originally isolated in 2003 (31) and previously draft sequenced as part of the Broad Institute’s *Enterococcus* Initiative, was PacBio sequenced (i.e., sequenced using Pacific BioSciences’ single-molecule real-time sequencing) and assembled to create a closed reference genome with improved plasmid resolution. The assembled genome consisted of a 2,717,680-bp circular chromosome, including 2,662 predicted coding sequences, as well as four plasmids (pAR01.1 to pAR01.4). Of significance to this work, two of these plasmids carry resistance to four agriculturally and clinically relevant antibiotics: bacitracin, vancomycin, tetracycline, and erythromycin. Most of the pAR01.1 sequence is not similar to other published plasmid sequences, but the other three plasmids have substantial similarity to widely occurring poultry and clinical E. faecalis plasmids. pAR01.2 is most similar to pTEF2 from the pathogenic E. faecalis strain V583 (42), while pAR01.3 is most similar to pVEF3, a plasmid originally isolated from Norwegian poultry farms (43). In both cases, plasmids share >99% sequence similarity over ∼60% of the plasmid length. pAR01.4 is most similar to pAMalpha1, which is associated with tetracycline resistance (44). In all these cases, the pAR01 plasmids are smaller than these reference plasmids.

Plasmid pAR01.1 (length, 65,136 bp) carries both tetracycline resistance determinants and the bacitracin resistance *bcr* operon. Tetracycline resistance is conferred by a gene encoding a tetracycline efflux protein (gene *tetL*) and an additional gene encoding a ribosome protection protein (*tetM*) (45, 46). Bacitracin resistance is provided by the products of a *bcr* operon, including a response regulator (*bcrR*), ABC transporter (*bcrA* and *bcrB*), and putative undecaprenyl diphosphatase (*bcrD*) (47–49). Notably, pAR01.1 also carries at least four independent plasmid addiction systems that have previously been implicated in plasmid maintenance. These include Rel, Maz, and Fst family toxin-antitoxin pairs, as well as a bacteriocin and the bacteriocin immunity protein (50).

Plasmid pAR01.3 (length, 31,392 bp) carries both a vancomycin resistance gene cluster and erythromycin resistance determinants. Vancomycin resistance is encoded by a cluster of seven genes (*vanR*-A, *vanS*-A, *vanH*-A, *vanA*, *vanX*-A, *vanY*-A, and *vanZ*-A) known collectively as the *vanA* genotype. Typically, VanA-type VRE are also resistant to similarly structured glycopeptides such as avoparcin and teicoplanin (51). The resistance genes *ermL* and *ermB* may confer resistance to other macrolides such as lincosamides and streptogramin B in addition to erythromycin (52). In contrast to pAR01.1, pAR01.3 carries a single toxin-antitoxin system (Fig. S7).

### Multiple antibiotic resistance is common within both New Zealand clinical and poultry VRE.

**FIG 3:**
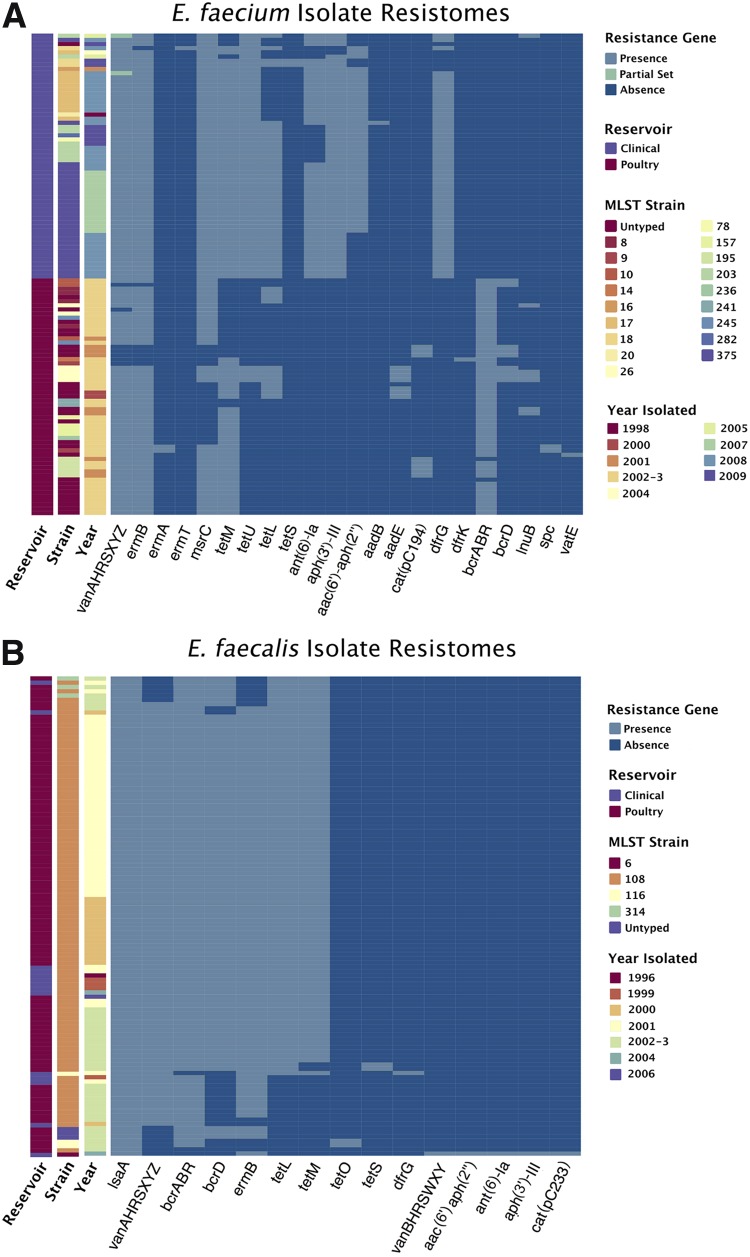
Antibiotic resistance genes detected in vancomycin-resistant E. faecalis and E. faecium isolates. The colors indicate the presence (light blue) or absence (dark blue) of each resistance gene in the genome of each E. faecium (A) and E. faecalis (B) isolate. Vertical bars (left) indicate the sample reservoir, MLST strain, and year of isolation of each isolate. Resistance genes are identified by commonly used abbreviations for simplicity.

The assembled contigs for all sequenced E. faecalis and E. faecium isolates were screened for antimicrobial resistance genes using the Resfinder database (53), and BLAST was used to screen for additional antibiotic resistance genes that were not in this database (*qac* and *bcr*) (see Materials and Methods). A variety of resistance genes were detected (Fig. 3; Table S4, Table S5, and Fig. S8).

### Resistance patterns in *E. faecium* show higher heterogeneity and diverse, reservoir-specific resistance genes.

As expected, the phylogenetically diverse collection of E. faecium isolates had corresponding heterogeneous antibiotic resistance gene profiles. The majority of both poultry and clinical isolates carried resistance genes for vancomycin (93.1%), erythromycin/macrolides (95.7%), and tetracycline (85.3%). Poultry isolates contained the bacitracin resistance *bcr* operon (96.5%), but this was absent in all clinical isolates (Fig. 3A). Clinical isolates carried additional resistance genes for tetracycline (*tetU*, 100%), aminoglycosides [*ant(6)-la*, *aph(3′)-III*, *aac(6′)-aph(2″)*, 100%], and trimethoprim (*dfrG*, 89.8%). This observed reservoir of resistance gene carriage likely reflects different exposure to antimicrobials in these settings and further emphasizes the separation between the clinical and poultry reservoirs in E. faecium.

### *E. faecalis* resistance patterns show high uniformity and support a poultry origin for an abundant clinical VRE clone.

The antibiotic resistance gene profiles of sequenced E. faecalis isolates showed high homogeneity. The majority of isolates carried a consistent set of resistance genes, predominantly variants of vancomycin (90%), erythromycin (90.9%), tetracycline (84.0%), bacitracin (71.9%), and clindamycin (51.9%) resistance (Fig. 3B). One clinical E. faecalis strain was unique, carrying a *vanB*-type vancomycin resistance operon; this was the only E. faecalis isolate with aminoglycoside and chloramphenicol resistance genes within the data set. All 13 clinical isolates with bioinformatically detected bacitracin resistance genes belonged to the ST108 lineage and carried a variation of the *bcrRABD* operon sequence (sometimes lacking *bcrD*). To verify clinical bacitracin resistance, PCR amplification of the *bcrR* gene from culture genomic DNA and bacitracin MIC testing of isolates were performed. Of the 13 isolates, 12 were PCR positive for the *bcrR* gene and bacitracin resistant at ≥256 μg ml^−1^. The exceptions were strain AR99-1107, which was phenotypically bacitracin resistant but PCR negative for *bcrR*, and AR00-128, which was PCR positive for *bcrR* but bacitracin sensitive (32 μg ml^−1^) due to a truncated *bcrB* gene (Table S6).

Because some isolates carried multiple copies of the bacitracin resistance genes, uclust (54) was used to cluster bacitracin resistance genes into alleles. Two distinct sets of bacitracin resistance genes were revealed. The first had 100% sequence identity to the *bcrRABD* operon previously reported from E. faecalis isolate AR01/DG (31). The second operon comprised the *bcrRAB* genes, each of which had approximately 90% sequence identity to the genes identified in AR01/DG. Isolates within the data set contained either one or (predominantly) both sets of *bcr* genes, but either set alone was sufficient to confer full phenotypic bacitracin resistance (Table S6, Fig. S9), and both sets of homologs were present in both clinical and poultry isolates.

### Vancomycin resistance is sometimes lost in the absence of selective pressure.

A subset of VRE isolates had previously demonstrated vancomycin resistance in culture and had phylogenetic relationships with VanA-positive isolates. Therefore, they were expected to carry the *vanA* genotype, but neither the *vanA* nor *vanB* vancomycin resistance cassettes were initially detected in the assembled contigs by ABRicate or BLAST, nor were these gene sequences detectable in unassembled reads using bowtie2. Therefore, PCR was used to amplify the *vanA* gene from culture genomic DNA, and the vancomycin MIC of the isolates was reverified. A comparison between the original isolation glycerol stocks and the secondary sequencing glycerol stocks indicated that in all but one case (strain TV147), vancomycin resistance was lost during growth in nonselective media immediately prior to sequencing (Table S7). Notably, in 17 of the 19 cases, loss of previously lab-verified vancomycin resistance in the sequencing data was accompanied by the absence of plasmid colocalized *ermB* resistance genes for strains with previously lab-verified erythromycin resistance, further supporting loss of this plasmid. This highlights the ability of some strains to lose a plasmid containing vancomycin and erythromycin resistance genes (e.g., pAR01.3) in the absence of constant selective pressure, in contrast to previous reports (29). Within ST108, there were three instances of spontaneous vancomycin resistance loss immediately prior to sequencing, but only one instance of bacitracin resistance loss immediately prior to sequencing.

### A role for coselection in the stability of VRE in New Zealand agriculture.

Two of the four plasmids (pAR01.1 and pAR01.3) in the AR01/DG genome are multidrug resistance plasmids with colocalized resistance genes. Colocalization provides multiple mechanisms (e.g., use of more than one antibiotic) to ensure plasmid maintenance and has been previously observed to stabilize plasmids in VRE (55, 56). However, plasmid content is much less stable than chromosomal content. In particular, approximately two-thirds of pAR01.1 and pAR01.3 plasmid content is conserved in over 80% of ST108 isolates, while approximately 85% of all plasmid content is conserved in ∼50% of ST108 isolates (Fig. S10).

**FIG 4:**
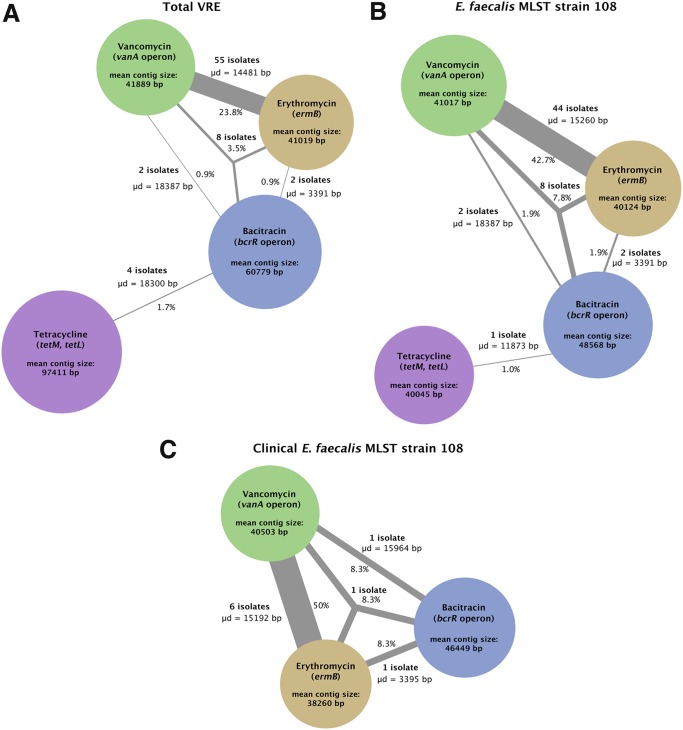
Colocalization of selected antibiotic resistance genes on assembled contigs across isolates. Network diagrams indicate the number of isolates for which bacitracin, erythromycin, tetracycline, and/or vancomycin resistance genes were colocalized on assembled contigs. Results are shown for all VRE isolates (A), all E. faecalis ST108 isolates (B), and all clinical E. faecalis ST108 isolates (C). Colored circles indicate different antibiotic resistance genes (green, vancomycin; blue, bacitracin; brown, erythromycin; purple, tetracycline). The circle diameter corresponds to the average size of contigs containing the relevant resistance gene colocalized with another resistance gene(s). Lines between these circles link resistances found on a single contig and are labeled with the average distance between the antibiotic resistance genes, the numbers of isolates in which the resistance genes were contig colocalized, and the percentage of total isolates within the relevant isolate set in which this colocalization was found.

To better quantify the extent of colocalization of resistance determinants in NZ VRE, we analyzed the assembled contigs of all VRE isolates for colocalization of the most common resistance determinants (*van*, *erm*, *bcr*, and *tet*). In E. faecalis, the most commonly observed colocalization was of the *vanA* operon with *ermB*, observed in 42.7% of the total E. faecalis strain 108 isolates (44 isolates), and 50% of the clinical E. faecalis strain 108 isolates (6 isolates) (Fig. 4, Table S8). This is consistent with the colocalization of the *vanA* operon and *ermBL* on pAR01.3 in the PacBio-finished reference genome.

However, contig colocalization was often inconsistent with AR01/DG plasmid arrangement. For instance, we observed 10 isolates of contig colocalization of the *vanA* operon and *bcrRABD* genes and 10 isolates with contig colocalization of *ermB* and *bcrRABD*. These instances included both clinical and poultry isolates (Table S8). In addition, *tetLM* and *bcrRABD* were contig colocalized in four isolates, one of which belonged to ST108. Notably, there were eight instances, including poultry and clinical isolates, in which the *vanA* operon and the *ermB* and *bcrRABD* genes were all present on the same contig. This was observed only in E. faecalis ST108 isolates. These data suggest that the resistance plasmids found in New Zealand VRE may be recombinant, and ongoing evolution of plasmids has clinical relevance.

NZ E. faecium carried a markedly different set of resistance determinants than E. faecalis. The most commonly observed colocalization was the aminoglycoside resistance genes *ant(6)-la* and *aph(3′)-III* with *ermB*, occurring in 37% of the total E. faecium isolates (43 isolates). This was expected because these genes also colocalize in plasmid pE1_3 of the genome of E. faecium E1, a close relative to many of these isolates. We also observed 10 E. faecium isolates with the *tetM* and *dfrG* resistance genes on the same contig, suggesting colocalization. Interestingly, these colocalizations were only observed in E. faecium clinical isolates.

## DISCUSSION

Previous studies have suggested a causative link between the agricultural use of antibiotics and the emergence of vancomycin-resistant E. faecalis in a clinical setting in NZ (29–31). Whole-genome sequencing of both NZ clinical and agricultural vancomycin-resistant E. faecalis isolates in this study enabled the construction of a detailed phylogeny, as well as analysis of antibiotic resistomes within this phylogenetic framework. Both the phylogeny and the distribution of antibiotic resistance genes suggest that a highly persistent and multidrug-resistant E. faecalis strain, ST108, has spread from poultry to human reservoirs. In addition, colocalization of antibiotic resistance determinants suggests that the continued agricultural use of nonvancomycin antibiotics in NZ could contribute to the maintenance of vancomycin resistance within enterococci, despite the discontinuation of avoparcin to reduce VRE prevalence.

### Historical context of samples in this study.

All clinically isolated VRE in NZ are collected at the ESR for sequence typing and monitoring. VRE were not observed in a clinical context in NZ until 1996 and were initially observed in low numbers (i.e., <10 clinical isolates per year). Almost all clinical isolates collected between 1996 and 2007 were VanA-type E. faecalis; the first NZ hospital-associated outbreaks of vancomycin-resistant E. faecium (both VanA and VanB) occurred in 2007 and 2008 (Table S3). Vancomycin-resistant E. faecium has subsequently dominated in NZ hospitals, with 70 to 100 clinical isolates observed per year.

Poultry isolates in this study came from two previous studies of NZ poultry. The first study densely sampled eight farms from a single major poultry supplier over a limited geographic area (29). The second study more shallowly sampled 147 farms from three major suppliers and three geographic areas, including both the North and South Islands (Table S1), and is therefore more broadly representative of the ∼180 commercial poultry farms in NZ (30). Vancomycin-resistant E. faecium represented 15% of sequenced poultry isolates from 2000 to 2001 but 55% of sequenced poultry isolates from 2002 to 2003. Most of this difference is likely due to sampling differences between studies, particularly the broader geographic range of the study from 2002 to 2003 compared to the study from 2001 to 2002. The predominance of E. faecalis ST108 in both studies indicates that it was a common poultry VRE strain during the study period. These data further suggest persistence of VRE in NZ agriculture at least 6 years beyond the period where avoparcin was in use, since ST108 VRE were isolated as late as 2004 clinically and 2006 agriculturally (Fig. S4).

An important strength of this study is that it includes sequencing data from almost all clinical cases of VRE in NZ in the surveyed period (1996 to 2009). However, one limitation is that most cases of vancomycin-resistant E. faecium in NZ have only occurred since 2004, outside our collection window of poultry isolates. Many clinical cases of vancomycin-resistant E. faecium in NZ are also associated with previous stays in overseas hospitals and linked to global vancomycin-resistant E. faecium strains belonging to lineages associated with nosocomial transmission and outbreaks (57). Further work is required to fully understand the epidemiological linkages of vancomycin-resistant E. faecium cases in NZ.

### Clonal expansion of vancomycin-resistant *E. faecalis* across reservoirs.

The phylogeny generated in this study of recombination-corrected core genome alignments of the E. faecalis ST108 type showed clinical isolates interspersed within and between poultry isolates, in contrast to NZ vancomycin-resistant E. faecium isolates. Clinical isolates were separated from poultry isolates by as few as 10 SNPs and often shared the same number of SNP differences from poultry isolates and clinical isolates (Fig. S4). Raven et al. (58) have previously calculated an E. faecalis evolution rate of 2.5 to 3 SNPs per genome per year, while the rate of evolution we estimated with BactDating (1.5 SNPs per genome per year) was slightly lower. Direct consideration of these estimates suggests that transmission does not occur between poultry and clinical cases in this study. However, it is important to note that phylogenetic diversity within a given poultry sample is within the range of what we observe between clinical and poultry isolates; therefore, transmission between poultry and human reservoirs is plausible. Furthermore, bacitracin resistance (either phenotypically or genetically detected, and usually both) was nearly ubiquitous in all poultry VRE isolates (both E. faecalis and E. faecium), but within clinical isolates it was exclusive to and ubiquitous within the ST108 lineage. This is consistent with a transfer to a human reservoir after acquisition of bacitracin resistance genes in an agricultural setting. Finally, the fact that a few of our isolates spontaneously lost their resistance to vancomycin, erythromycin, or bacitracin when grown briefly without antibiotics in lab demonstrates that these genes are not inevitably stable within isolates over long periods of time. Rather, their continued persistence is more likely in the context of frequent antibiotic exposure. Collectively, these results suggest that the clinical ST108 isolates in this study are the result of transfer from agricultural to clinical settings rather than a transfer from clinical to agricultural settings.

Both historical and ongoing use of oral bacitracin in poultry farming contrasts markedly with the limited use of this antibiotic in human medicine, in which it is used topically but not orally (59, 60). The earliest isolation of ST108 was in NZ during a period when avoparcin, tylosin, bacitracin, and tetracycline were all concurrently available for use as growth promoters in agriculture; in 1999, NZ sales for avoparcin, macrolides, bacitracin, and tetracycline were 1,060, 6,082, 10,905, and 2,311 kg, respectively (59). Furthermore, the fact that 8% of our VRE isolates (and 3% of our ST108 isolates) lost vancomycin resistance when grown without vancomycin immediately prior to sequencing indicates that in some isolates, these resistance genes may be readily lost in the absence of constant selective pressure. Finally, the horizontally acquired resistance genes within the ST108 lineage correspond to three widely used agricultural antibiotics within this time period, which is consistent with a primary reservoir in NZ agriculture with maintenance by ongoing gastrointestinal exposure prior to 2000. According to the PubMLST database (61), the ST108 strain has only been observed in NZ (as early as 1996) and Malaysia (as early as 2005) (62). These observations are consistent with those made by initial studies in the early 2000s (29, 30) during the original investigation of these isolates. Both initial acquisition and maintenance of resistance to vancomycin, erythromycin, bacitracin, and tetracycline within ST108 are readily explained in an agricultural context. In contrast, tetracycline is the only one of these antibiotics that is routinely used clinically on a long-term basis, clinical vancomycin use is relatively uncommon, and bacitracin is only used topically. Initial acquisition and maintenance of the resistance determinants in the human clinical context therefore seem much less likely.

The high degree of phylogenetic similarity between ST108 isolates over a wide geographic sampling during the 2002-2003 sampling period may indicate a common vector for spread throughout poultry farms in NZ (Fig. 1). Possible vectors could include a breeder or feed manufacturer shared by many different poultry farms (30, 63). Alternatively, the clone may have spread throughout NZ decades ago and subsequently been retained by poultry housing, reinfecting each new flock and spreading between animals (30). Extended epidemiological investigation of isolates may be difficult due to the currently decreased prevalence of ST108 in NZ. There is, however, reason to believe that a stable reservoir for this clonal strain exists, since NZ clinical isolates matching the ST108 PFGE pattern have been isolated as recently as 2014 and 2017 (D. A. Williamson, unpublished data). This suggests that the ST108 lineage still has clinical relevance, and an investigation of its persistence within poultry is warranted. A modern investigation of NZ commercial poultry fecal samples would establish this lineage’s persistence within poultry, with a focus on breeder facilities, farm sheds, and poultry feed potentially illuminating how it spreads. The results of this study provide a high-resolution phylogenetic analysis of the structure of historical NZ VRE strains and a rationale for the development and spread of a clonal lineage, ST108, from poultry to clinical reservoirs on multiple occasions, as well as evidence for vancomycin coselection mechanisms.

### Pangenome and horizontal gene transfer.

The core genome (genes present in >95% of isolates) of ST108 comprised 2,503 genes. Regardless of whether the genome was analyzed on a per-gene basis or a sequence basis, the data consistently showed that the chromosomal genome, which contained 98.8% of core genes, was highly conserved between ST108 isolates, but plasmids, which contained 1% of core genes, were much less conserved across all isolates. Many low-frequency genes were not present in reference strain AR01/DG and therefore could not be classified as chromosomal or plasmid genes. The same pattern is apparent from analyzing conservation of 1-kb sequences within ST108, which showed that not all plasmids or portions of plasmids are equally conserved within the lineage. The phenomenon of ongoing plasmid rearrangement and recombination was further highlighted through analysis of colocalization of antimicrobial resistance genes, with varied colocalizations across the ST108 lineage. The contig-based analysis used in this study can provide only a conservative estimate of antibiotic coresistance, but long-read sequencing of plasmids found in VRE isolates could be used to provide a more comprehensive view of the frequency of antibiotic resistance cooccurrence within plasmids. A comprehensive plasmid analysis could also provide insight into the variety of resistance plasmids occurring across E. faecium strains or whether these have transferred between enterococcal species or other clinically significant bacteria.

One of the themes of this study is the significance of horizontal gene transfer as a driver of evolutionary diversification in NZ VRE. As illustrated, horizontally acquired plasmids carry a range of antimicrobial resistance determinants, of both clinical and agricultural relevance. These elements are also shown to carry known and hypothesized virulence genes, and appear to be readily rearranged, facilitating new coselection mechanisms and creating variation with possible selective advantages.

### Conclusions.

A clonal lineage of vancomycin-resistant E. faecalis dominated NZ poultry and clinical VRE isolations shortly following avoparcin discontinuation. The phylogeny and antibiotic resistance profile of this lineage are consistent with an origin in poultry during a period of avoparcin, tylosin, and bacitracin exposure, followed by spread multiple times into the human population. Although its incidence has since decreased, this clone persisted as late as 2006 within this study and is indicated to have clinically resurfaced in 2014 and 2017 (Williamson, unpublished). We suggest here that the coassociation between vancomycin, erythromycin, and/or bacitracin resistance genes and heavy antibiotic use in poultry industry in the study time period has contributed to the persistence of this lineage.

## MATERIALS AND METHODS

### Data source.

The 157 poultry VRE isolates sequenced and analyzed in this study were collected from various poultry farms across New Zealand under contract from three major companies over the 2000-2003 time period (29, 30) (Fig. S1). In 2000, 18 isolates were obtained from a pool of 66 broilers from poultry supplier A (29). In 2001, 55 isolates were obtained from 40 fecal samples received in equal proportions from four different farms of poultry supplier A (29). Over the course of a year from February 2002, 147 fecal samples, each from individual farms contracted by one of the three major companies, were collected; of these, a total of 77 VRE isolates were cultured. Metadata linking specific isolates to specific geographic locations were limited for this study (30). Isolates were cultured from glycerol freezer stocks overnight in brain heart infusion (BHI) media at 37°C prior to sequencing. Poultry isolate ARL08-192 was not collected during these prior studies but was isolated in 2006 and obtained by the ESR (New Zealand) in 2008.

The 74 clinical VRE isolates sequenced and analyzed in this study were obtained from an archive held by the ESR. These were obtained between 1998 and 2009 from various body sites (drain fluid, urine, abdominal wound, feces, tissue blood clot, rectal or perianal swab, ulcer, abscesses, continuous ambulatory peritoneal dialysis (CAPD) fluid, ileostoma, stoma, and bile aspirate) and were frequently isolated during screening procedures.

Metadata and an overview of sampling for all VRE isolates can be found in Table S1 and Fig. S1.

### Whole-genome sequencing of bacterial isolates.

All study isolates underwent whole-genome sequencing (WGS). DNA libraries were prepared using the Nextera XT DNA preparation kit (Illumina), and 2 *×* 150-bp paired-end sequencing was performed using the NextSeq platform (Illumina), as previously described (64). One isolate (AR01/DG) underwent genomic DNA extraction with the GenElute bacterial genomic DNA kit (Sigma-Aldrich), followed by sequencing on the RSII (Pacific Biosciences) with P6-C4 chemistry according to the 20-kb template preparation using the BluePippin size selection system protocol (Pacific Biosciences).

### Analysis of 231 isolate genomes using Nullarbor.

For individual isolate analysis, we used the Nullarbor pipeline (35) and associated software operations to perform quality control (Trimmomatic) (65), identify species (Kraken) (66), assemble contigs (SPAdes) (67), annotate genomes (Prokka) (68), perform MLST (69), determine antibiotic resistance profiles (abricate) (70), and detect SNPs relative to a reference genome (Snippy) (71). Species assignment was confirmed using pyANI (72) (Fig. S11). For the phylogenetic analysis of isolate sets, this study used the Nullarbor pipeline and associated software operations to calculate core genome SNPs (Snippy-Core) (71). Gubbins (36) was used to determine regions of genetic recombination and calculate a phylogenetic tree using RAxML. Snp-Dists (73) was used to calculate pairwise distances between isolates after Gubbins correction. Roary (74) was used to calculate the pangenome from annotated contigs. Nullarbor was applied to the sequenced whole-genome reads from several sets of isolates as indicated in Table 1 . Complete Nullarbor quality control data are available in Table S9.

**TABLE 1:** Isolate sets analyzed by Nullarbor

Set description	No. of isolates	Reference
All VRE isolates	231	E. faecalis CLB21560 (RefSeq accession no. NZ_CP019512.1)
All E. faecalis isolates	113	E. faecalis CLB21560 (RefSeq accession no. NZ_CP019512.1)
E. faecalis ST108 isolates	103	E. faecalis AR01/DG (NCBI collection: BioSample accession no. SAMN07371835, BioProject accession no. PRJNA395129)
All E. faecium isolates	116	E. faecium E1 (RefSeq accession no. NZ_CP018065)
E. faecium isolates without outliers AR98-565 and ARL09-409	114	E. faecium E1 (RefSeq accession no. NZ_CP018065)

### Assembly and annotation of the AR01/DG genome.

The genome assembly of AR01/DG involved a preliminary assembly using Canu (75), followed by circularization and manual confirmation with the bioinformatic software Geneious (76). An Illumina-based correction was carried out to achieve a higher accuracy assembly, using the Geneious “Map to Reference” and “Find Variations/SNPs” features.

At this stage, an additional small plasmid (pAR01.4) was identified in the unmapped Illumina reads which was absent in the initial Canu-assembled contigs. The assembled genome was annotated using the NCBI Prokaryote Genome Annotation Pipeline (77).

### Additional detection of antibiotic resistance genes.

For targeted searches of antimicrobial resistance genes within isolates, Illumina sequence reads were assembled into contigs using SPAdes (67) and contigs were queried using local BLAST databases and blastn or blastx (78) for the following gene sequences and accession codes: *qacC* (NG_048040.1), *qacA* (ARS14487), *bcrA* (AAS78451.1), *bcrB* (AAS78450.1), *bcrR* (AAS78452.1), *bcrD* (AAS78449.1), *vanA* (NC_014726.1), *bceA* (from NZ *S. uberis*, closest NCBI sequence: WP_046390890.1), *bceB* (from NZ *S. uberis*; closest NCBI sequence, WP_046390889.1), *bceR* (from NZ *S. uberis*; closest NCBI sequence, WP_046390888.1), *bceS* (from NZ *S. uberis*; closest NCBI sequence, WP_037592159.1). Bowtie2 (79) was used to align raw sequencing reads to the *bcrRABD* reference sequences to confirm the BLAST results for bacitracin resistance genes. Samtools (80) was used to create a BAM file to examine the read coverage for *bcrB* in isolate AR00-128. Local BLAST databases were created, and assembled isolate sequences were queried using *vanRSHAXYZ*, *ermB*, *bcrRABD* operon, and *tetM/tetL* sequences extracted from the DG_00004 and DG_00005 plasmids of AR01/DG in order to determine instances in which resistance genes to two or more antibiotics were present on the same contig.

In order to cluster highly similar *bcrRABD* gene variants for subsequent protein alignment, bedtools (81) was used to extract *bcrRABD* genes from the assembled contigs of each isolate using BLAST output data. The extracted sequences were then grouped into centroids with 95% sequence similarity using UCLUST (54), resulting in nine clusters of sequence variants, seven of which occurred in at least three isolates. All FASTA sequences within each cluster were then placed into the same 5′–3′ orientation using MAFFT (82). MUSCLE (83) was then used to align each cluster and determine conserved sequence positions within each gene.

### Calculation of sequence conservation across strains.

BLAT (84) was used to align the contigs from the 103 SPAdes-assembled E. faecalis ST108 isolates to the closed AR01/DG plasmid sequences (NCBI nucleotide collection for complete genomes: BioSample accession no. SAMN07371835 and BioProject accession no. PRJNA395129). The best alignments from BLAT outputs were parsed into BED files using a publicly available script created by Dave Tang (https://gist.github.com/davetang/7314846), and bedtools was then used to count the number of overlapping 1-kb regions with the AR01/DG plasmid reads, with a focus on the plasmids containing vancomycin and bacitracin resistance genes (pAR01.3 and pAR01.1).

### Dating of bacterial strains.

The BactDating R package (40) was used to estimate node dates in the E. faecalis and E. faecium phylogenies. Recombination-corrected alignments from Gubbins were used as input. BactDating used a mixed gamma model. A total of 2 *×* 10^7^ Markov chain Monte Carlo (MCMC) chains ensured an effective size of >100 for all estimated parameters, and traces were inspected to ensure mixing.

### Culture, susceptibility testing, and PCR confirmation.

For vancomycin susceptibility testing, poultry isolates with previously described vancomycin resistance in which vancomycin resistance genes were not bioinformatically detected were streaked on BHI agar plates from glycerol stocks drawn from for sequencing analysis and grown overnight at 37°C. Individual colonies were cultured overnight in BHI media at 37°C, and DNA extractions were performed on cultures using a Qiagen DNeasy blood and tissue kit according to the standard kit protocol for Gram-positive bacteria. MICs were analyzed by plating 1:10 dilutions of exponential-phase cultures in Mueller-Hinton (MH) broth on MH agar with bioMérieux Etest strips containing a gradient of vancomycin. If found to be *vanA* negative by PCR (FW, 5′-GTAGGCTGCGATATTCAAAGC-3′; RV, 5′-CGATTCAATTGCGTAGTCCAA-3′) and vancomycin susceptible by Etest, isolates were then recultured on 32 μg ml^−1^ vancomycin-enriched BHI plates from the original isolation glycerol stocks and subjected to analogous DNA extraction and MIC testing procedures.

For bacitracin susceptibility testing, clinical E. faecalis ST108 isolates were streaked onto BHI plates supplemented with 32 μg ml^−1^ bacitracin and cultured overnight at 37°C, as previously described (47). Individual colonies were cultured overnight in BHI media supplemented with 32 μg ml^−1^ bacitracin at 37°C, and DNA extractions were performed on cultures using the Qiagen DNeasy blood and tissue kit according to the standard kit protocol for Gram-positive bacteria. MICs were analyzed by plating 1:10 dilutions of exponential-phase cultures in MH broth on MH agar with bioMérieux Etest strips containing a gradient of bacitracin. DNA extractions were subjected to PCR using a primer set specific for one allele and a primer set specific for both identified alleles of *bcrR* (Set 1 FW, 5′-GGAAACTCTGTTGGCGGTTA-3′; Set 1 RV, 5′-ATGGTTCTCTTGCTGCTGCT-3′; Set 2 FW, 5′-CATTTACAGCCACCACACCA-3′; Set 2 RV, 5′-TTGCGACAAACAGCAGAAAC-3′).

### Data availability.

Data for this project are available in the NCBI database under BioProject accession no. PRJNA476469 and PRJNA395129. The source code is available at https://gitlab.com/morganx/vre2019.

## Supplementary Material

Supplemental file 1

Supplemental file 2
