# Supplementary material for: Agricultural Origins of a Highly Persistent Lineage of Vancomycin-Resistant Enterococcus faecalis in New Zealand
Source: Appl Environ Microbiol. 2019 Jun 17;85(13):e00137-19. doi: 10.1128/AEM.00137-19 (PMC6581176; doi:10.1128/AEM.00137-19)
Supplement: Supplemental file 1 [file AEM.00137-19-s0001.pdf]

Supplement

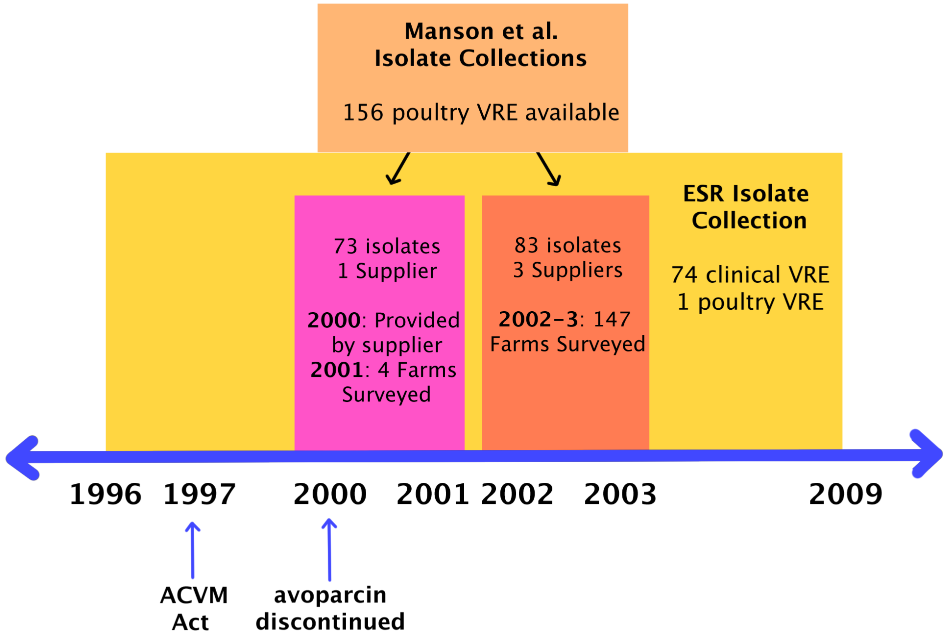

Figure S1. *Schematic diagram of sample collection*. The yellow and tan blocks represent the timespans in which the clinical VRE isolates in this study were collected by ESR and the poultry isolates were collected by Manson et al. respectively. Numbers of isolates in each collection are indicated. The purple and orange blocks represent the sampling periods for each of Manson et al's studies (2000-1: (1), 2002-3: (2)), and indicate the number of isolates, number of suppliers, and number of farms surveyed where known. The timeline also indicates when legislation was introduced to regulate antibiotic use in animal husbandry (1997) and when avoparcin was officially discontinued for sale in New Zealand (2000).

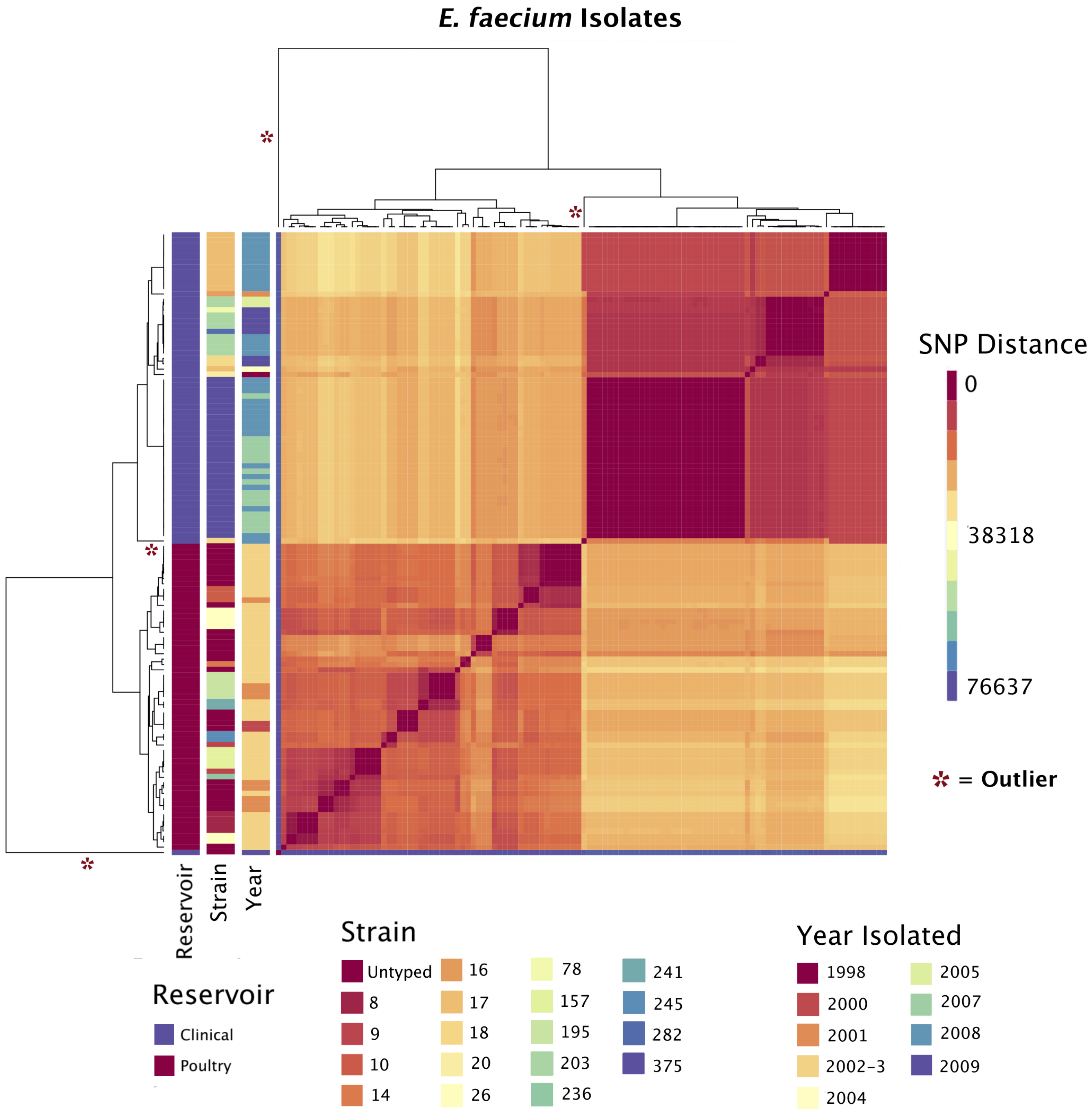

Figure S2. *Heatmap indicating SNP distances across the core alignments of the total E. faecium isolate set*. The core alignment comprises a clonal frame of 2,933,268 bp and contains 81,122 SNPs. Colors indicate SNP distances between isolates (center), reservoir (left vertical bar), MLST strain (center vertical bar) and year isolated (right vertical bar). The reference sequence is a clinical *E. faecium* strain E1 complete genome (NCBI RefSeq NZ_CP018065).

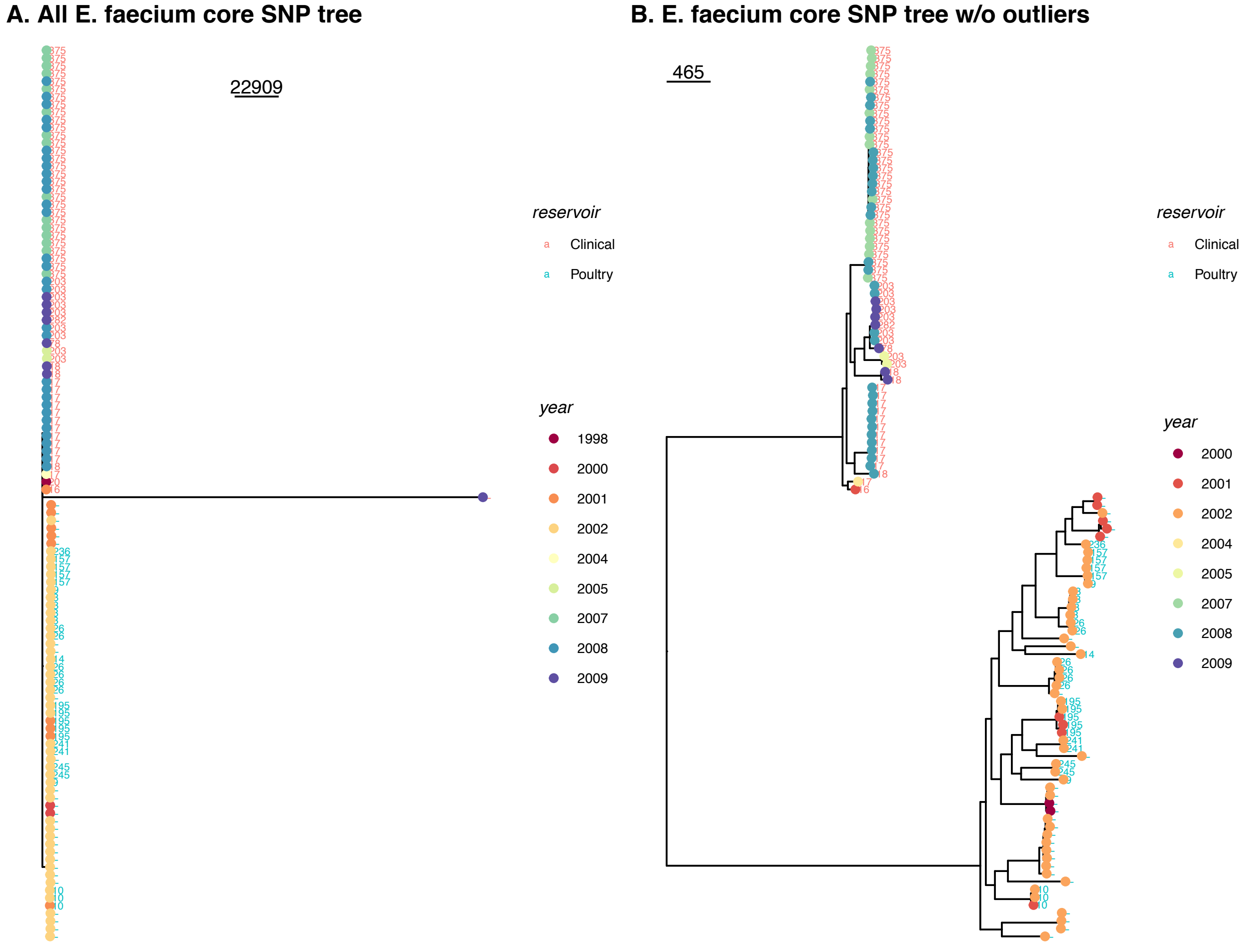

Figure S3. *Core phylogenetic trees of important E. faecium subsets.* A) Total vancomycin-resistant *E. faecium* isolates, phylogeny created from a core alignment containing 81,122 SNPs. B) Vancomycin-resistant *E. faecium* excluding outliers, phylogeny created from a core alignment containing 9,835 SNPs. Reservoir (clinical/poultry) is indicated by text color and collection year is indicated by circle color. Tip label text indicates MLST strain identification ("–" indicates unidentified MLST strain). The reference sequence is a clinical *E. faecium* strain E1 complete genome (NCBI RefSeq NZ_CP018065). Scale bar indicates number of SNPs. between strains.

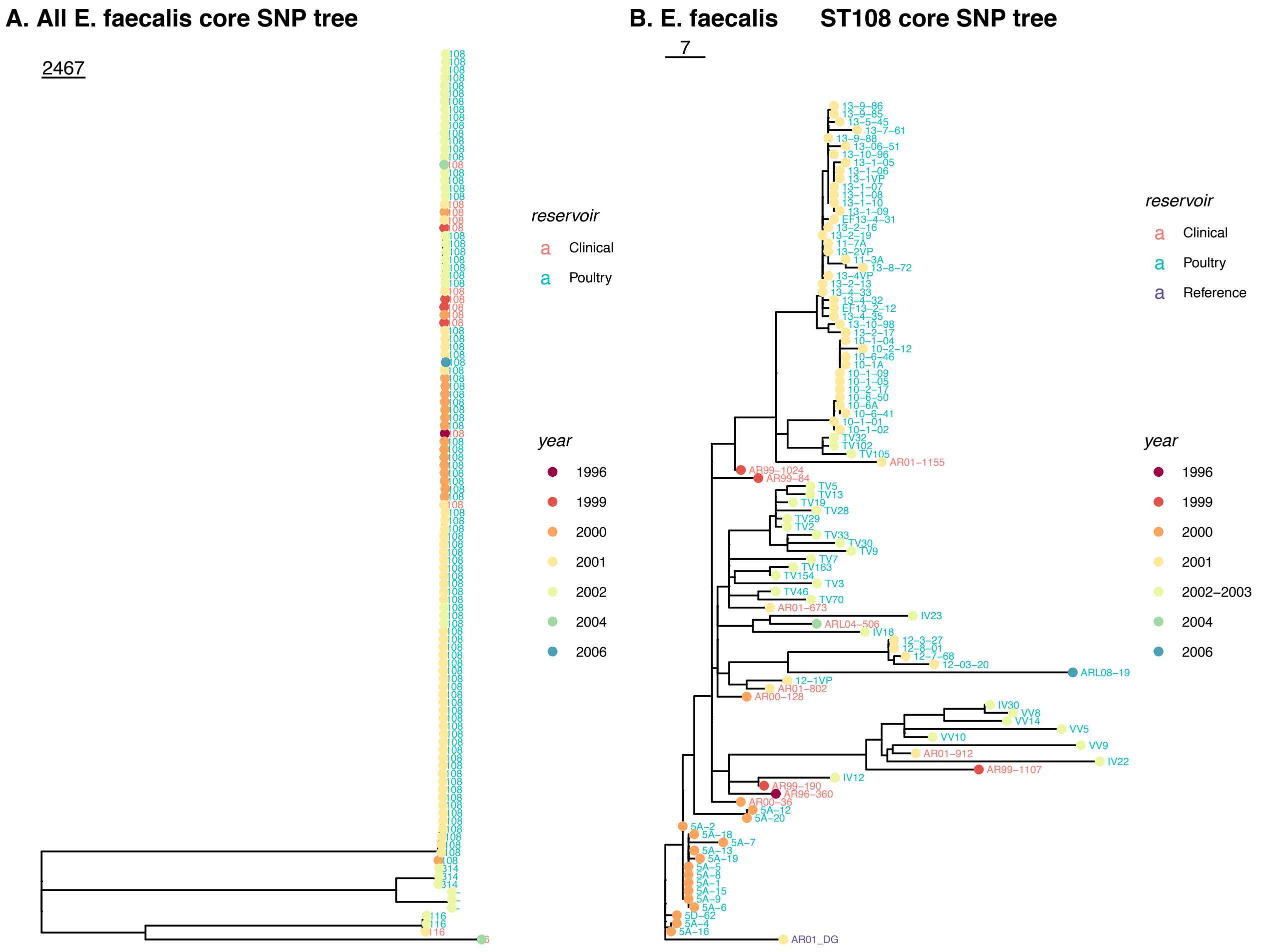

Figure S4. *Core phylogenetic trees of important E. faecalis subsets.* A) Total vancomycin-resistant *E. faecalis* isolates, phylogeny created from a core alignment containing 42,058 SNPs. B) Vancomycin-resistant MLST 108 *E. faecalis* isolates, phylogeny created from a core alignment containing 664 SNPs. Reservoir (clinical/poultry) is indicated by text color and collection year is indicated by circle color. Tip label text indicates A) MLST strain identification ("–" indicates unidentified MLST strain) and B) isolate ID. The phylogeny is indicated in both circular and linear formats. The reference sequences are a clinical *E. faecalis* strain CLB21560 complete genome (NCBI RefSeq NZ_CP019512.1) and an *E. faecalis* strain 108 complete genome isolated from a dog with mastitis (AR01/DG) respectively. Core alignments and SNP distances were derived using Nullarbor and Gubbins. Scale bar indicates number of SNPs.

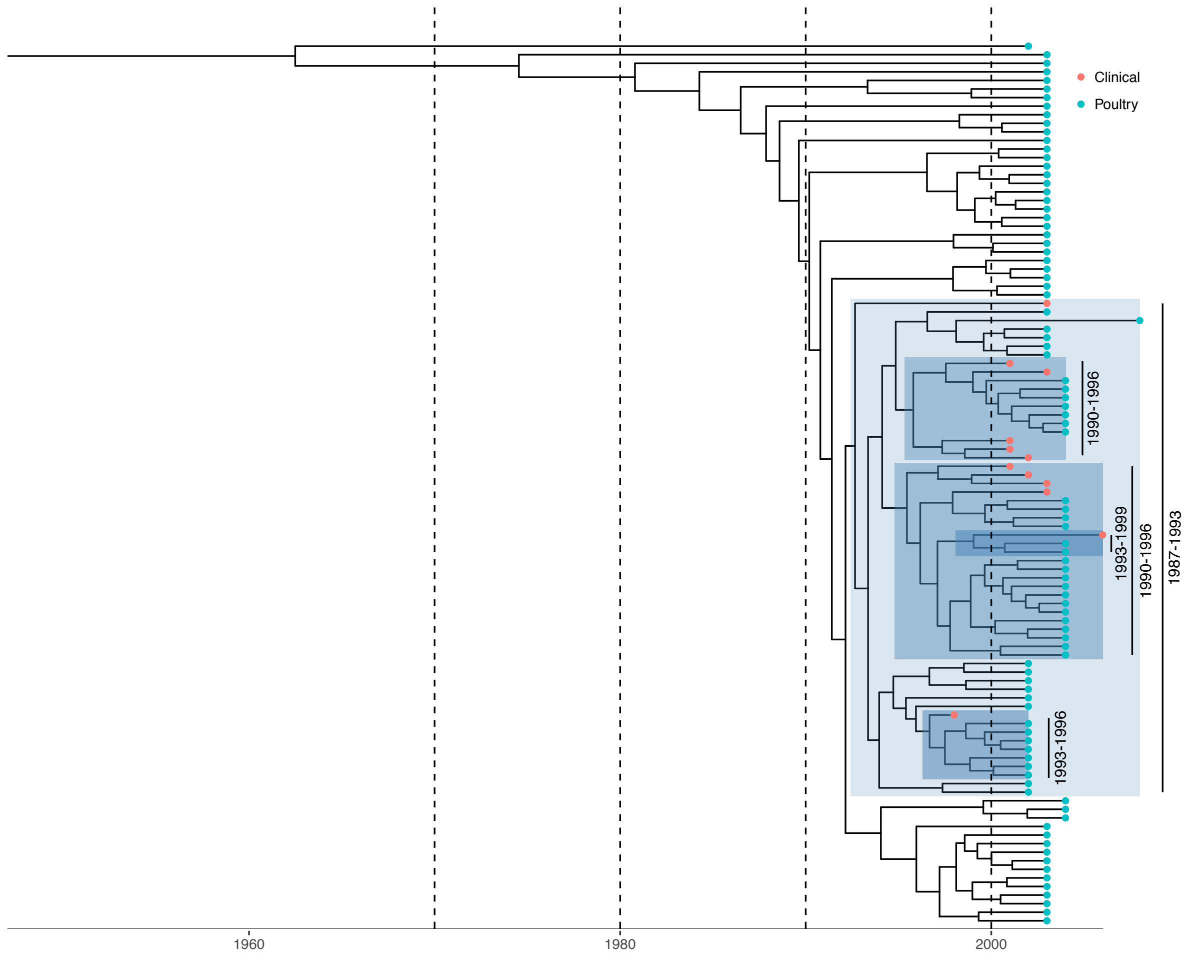

Figure S5. *Bayesian dating analysis of ST108.* Tip labels correspond to reservoir of each isolate. Shaded blue boxes highlight nodes of poultry/clinical divergence. Corresponding black lines indicate 95% credible intervals for node dates.

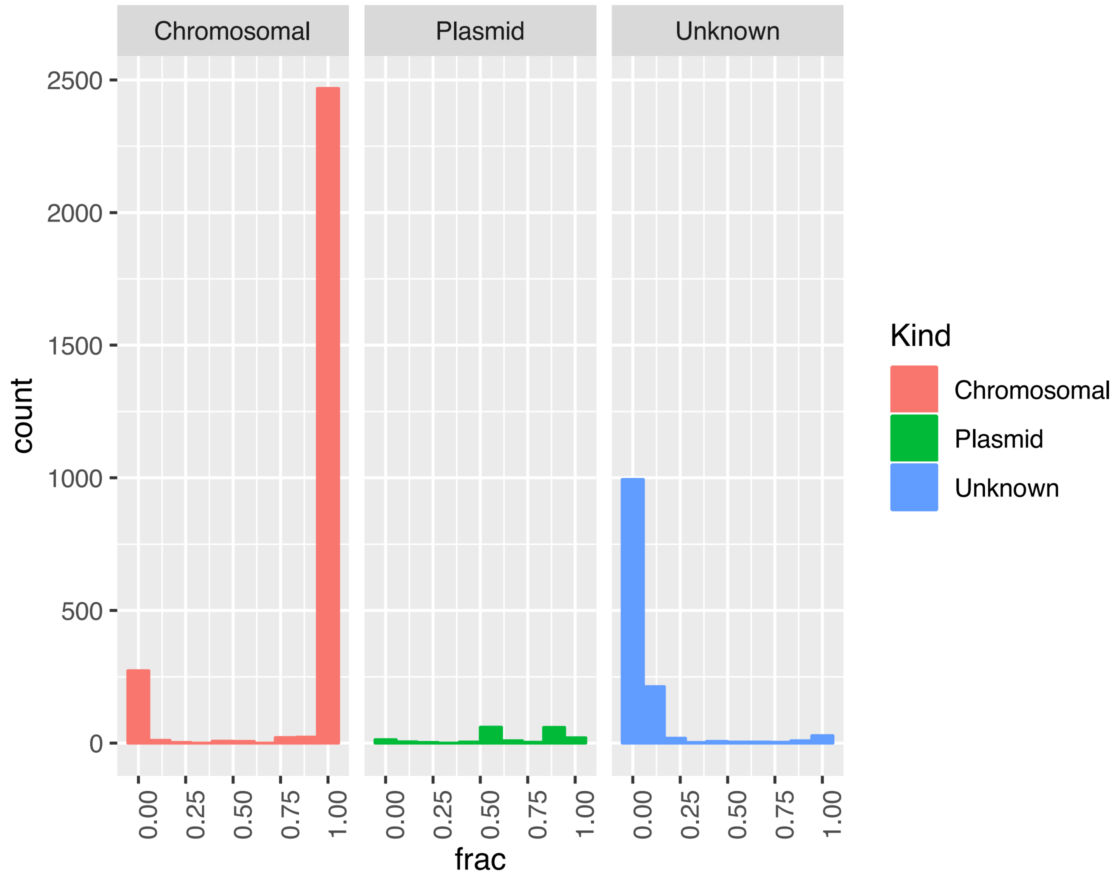

Figure S6: *Conservation of chromosomally and plasmid-localized genes across strains.* Roary was used to calculate conservation of genes across strains. The status of each gene was assigned as chromosomal (if on AR01/DG chromosome 1), plasmid (if on an AR01/DG plasmid), or unknown (if not present in AR01/DG). This figure shows histograms, in which the X axis corresponds to the total number of strains, and the Y axis corresponds to the total number of genes present in a fraction of strains.

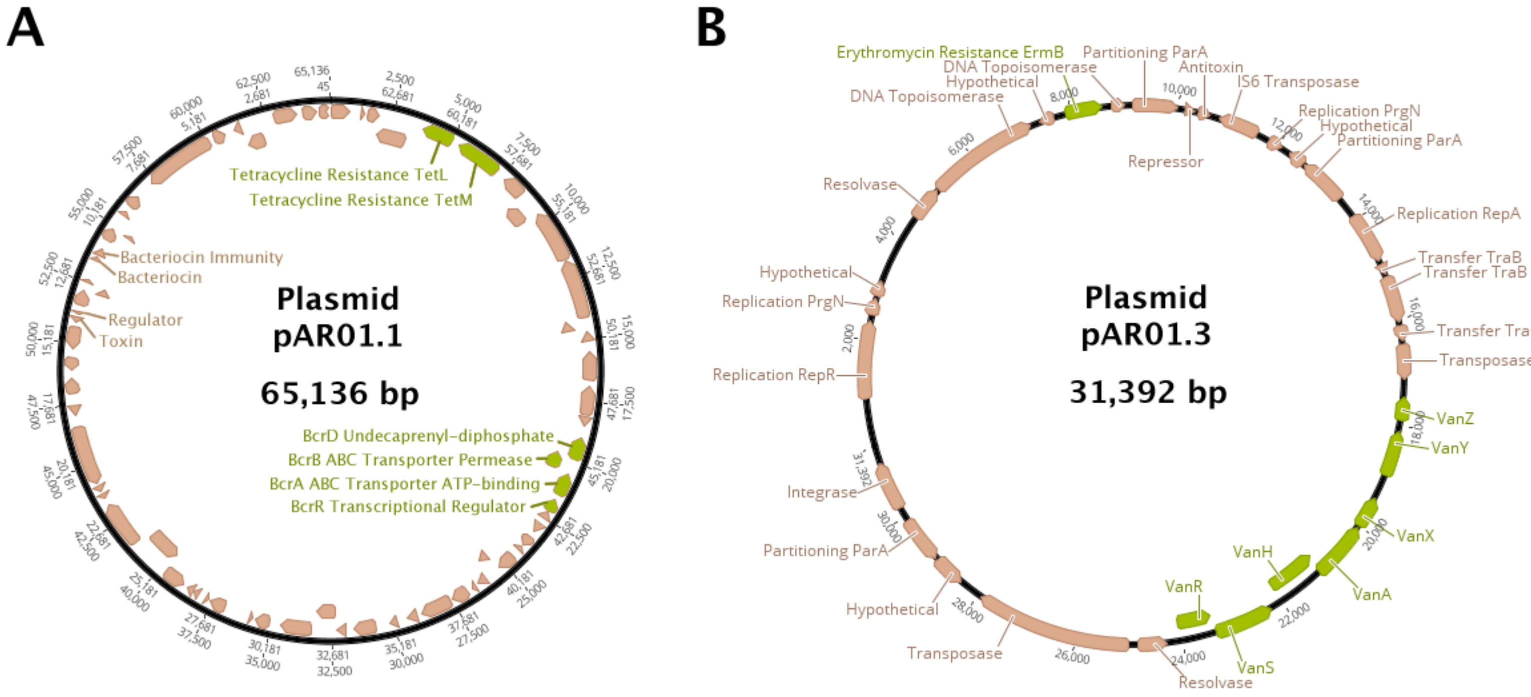

Figure S7: *Plasmids with antibiotic resistance elements in strain AR01/DG.* A) Plasmid map of pAR01.1 containing bacitracin and tetracycline resistance genes. B) Plasmid map of pAR01.3 containing vancomycin and erythromycin resistance genes. Plasmid annotation and visualization partially derived using Geneious.

Unidentified Species Isolate Resistomes

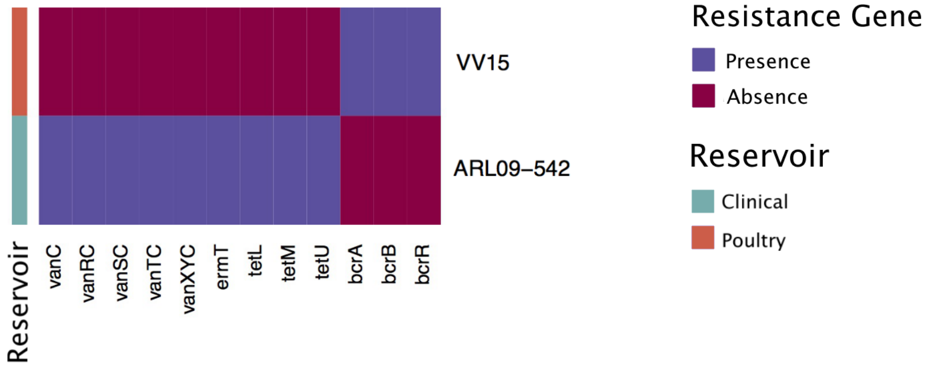

Figure S8. *Heatmaps of bioinformatically-detected resistance genes in unidentified species isolates.* Colours indicate presence/absence of each resistance gene in each isolate (centre), reservoir (left vertical bar), and year isolated (right vertical bar). Axis labels indicate resistance gene identification. Assembled contigs were checked for resistance genes using Nullarbor [ABRicate], BLAST and/or bowtie2.

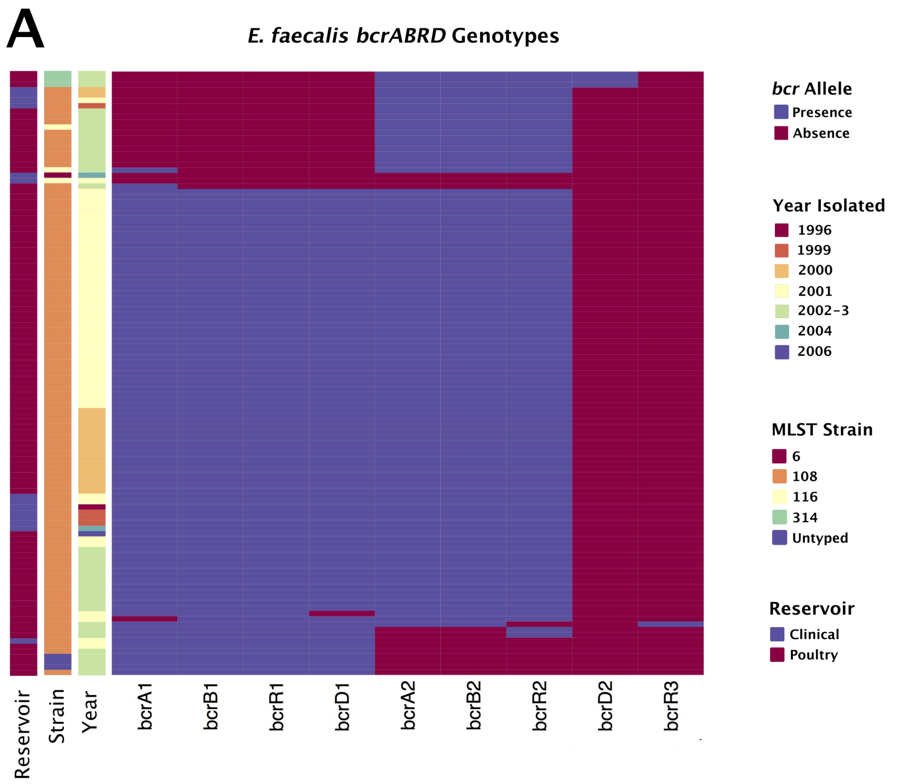

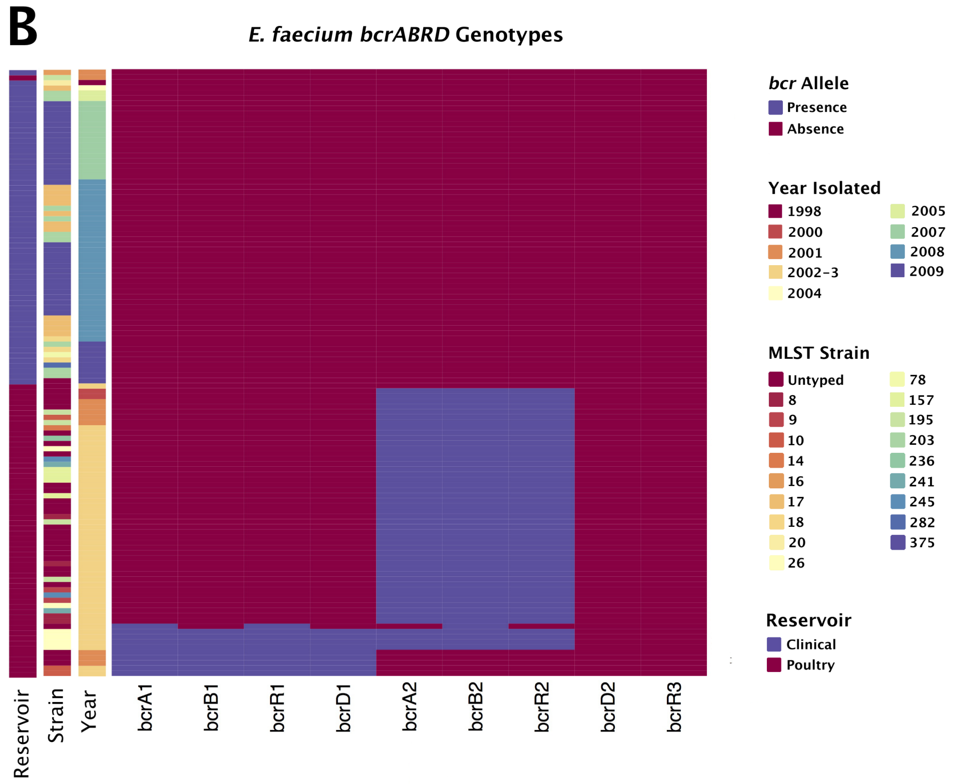

Figure S9. *Heatmaps of bacitracin resistance alleles found bioinformatically in (A) E. faecalis and (B) E. faecium isolates*. Colours indicate presence/absence of each allele in each isolate (centre), reservoir (left vertical bars), MLST strain (centre vertical bars) and year isolated (right vertical bars). Axis labels indicate bacitracin resistance allele identification (y axis text) and isolate codes (x axis text). Allele variants share ~90% sequence similarity across equivalent genes.

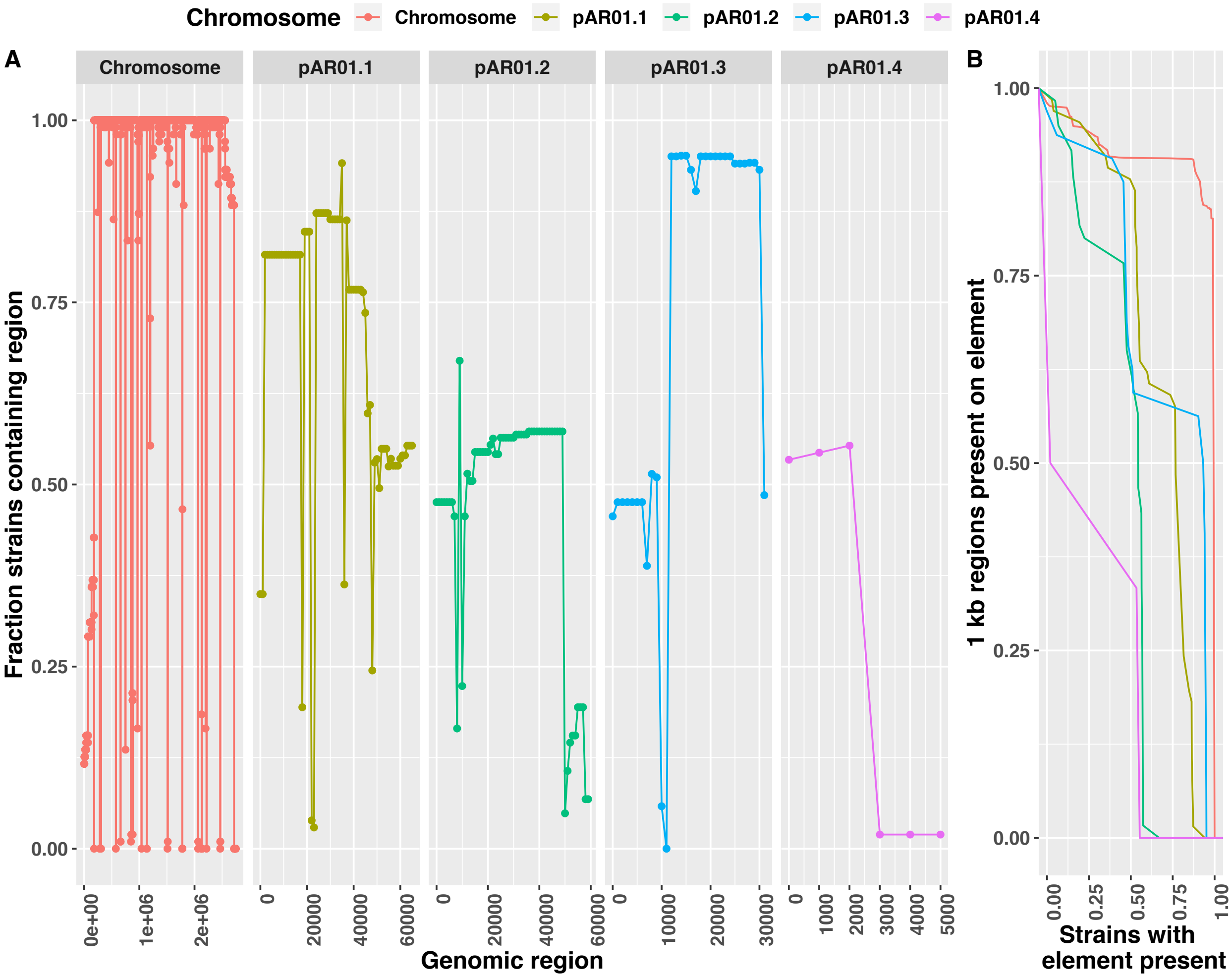

Figure S10. *Conservation of genetic elements across strains.* The genome of ARO1/DG was divided into 1kb regions, and BLAST was used to determine the presence of the region in other strains. (A) Each graph corresponds to a genetic element (chromosome or plasmid). The X axis corresponds to physical position on the element. The Y axis corresponds to the fraction of total MLST 108 isolates in which this 1kb element was present. (B) Empirical cumulative distribution of regions within strains. The X axis indicates the cumulative fraction of all MLST108 strains containing at least Y fraction of the total 1kb regions on each chromosome and plasmid. Color corresponds to chromosome and plasmid.

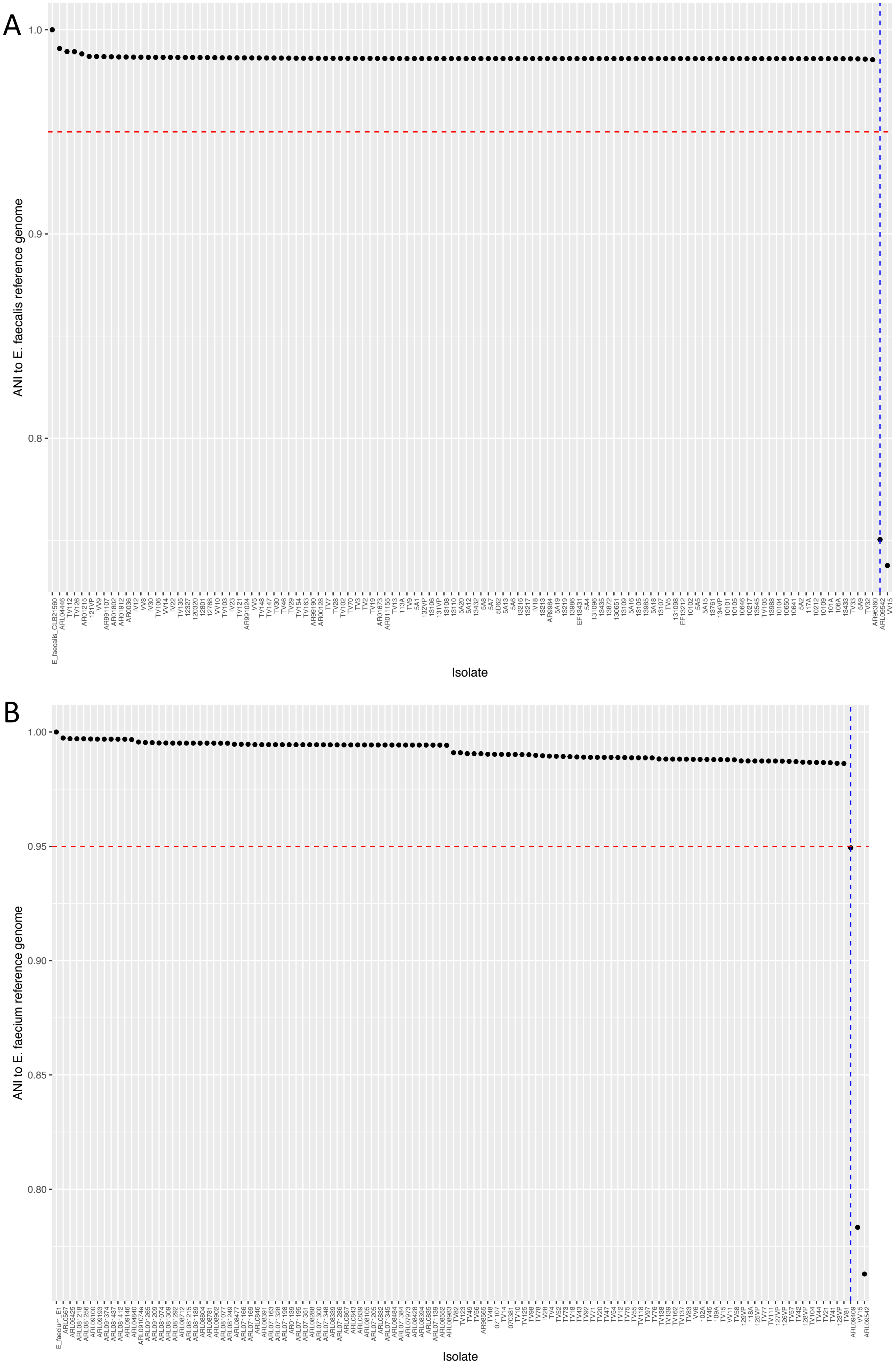

A
ANI to E. faecalis reference genome
1.0
0.9
0.8
Isolate
B
ANI to E. faecium reference genome
1.00
0.95
0.90
0.85
0.80
Isolate

Figure S11. Average nucleotide identity to reference genomes of isolates in study. A) Average nucleotide identity to reference genome *E. faecium* E1 of all isolates in this study classified as *E. faecium* or unclassified. The red horizontal line at 0.95 indicates the accepted ANI threshold for species identity, and the blue vertical line separates *E. faecium* isolates from non-*E. faecium* isolates. B) Average nucleotide identity to reference genome *E. faecalis* CLB21560 of all isolates in this study classified as *E. faecalis* or unclassified. The red horizontal line at 0.95 indicates the accepted ANI threshold for species identity, and the blue vertical line separates *E. faecalis* isolates from non-*E. faecalis* isolates.

References

1. Manson JM, Keis S, Smith JM, et al. A clonal lineage of VanA-type Enterococcus faecalis predominates in vancomycin-resistant Enterococci isolated in New Zealand. *Antimicrob Agents Chemother* 2003;47(1):204-10.
2. Manson JM, Smith JM, Cook GM. Persistence of vancomycin-resistant enterococci in New Zealand broilers after discontinuation of avoparcin use. *Appl Environ Microbiol* 2004;70(10):5764-8.
